# Change in Hemoglobin A1c and Quality of Life with Real-Time Continuous Glucose Monitoring Use by People with Insulin-Treated Diabetes in the Landmark Study

**DOI:** 10.1089/dia.2020.0666

**Published:** 2021-03-02

**Authors:** Timothy R. Gilbert, Adam Noar, Olivia Blalock, William H. Polonsky

**Affiliations:** ^1^ Endocrinology Center of Southwest Louisiana, Lake Charles, Louisiana, USA.; ^2^ Dexcom, Inc., San Diego, California, USA.; ^3^ Behavioral Diabetes Institute, San Diego, California, USA.; ^4^ Department of Medicine, University of California, San Diego, California, USA.

**Keywords:** Continuous glucose monitoring, Patient-reported outcomes, HbA1c

## Abstract

***Background:*** Initiating continuous glucose monitoring (CGM) can affect hemoglobin A1c (HbA1c) levels and patients' relationship with their diabetes. We used real-world HbA1c data to quantify short-term changes in glycemia and validated psychosocial questionnaires to assess changes in quality-of-life indicators in people during their first few months of CGM use.

***Methods:*** Eligibility was assessed during calls to Dexcom sales regarding its G6 CGM System. Eligibility criteria included ages 25–65 years, type 1 (T1D) or type 2 diabetes (T2D) on intensive insulin therapy (IIT), and no prior CGM use. Participants used a web-based portal to complete the 17-item Diabetes Distress Scale (DDS) and the 14-item Hypoglycemia Attitudes and Behavior Scale (HABS); provided validated HbA1c measurements; and shared their CGM data pre- and 3–5 months post-CGM initiation. Satisfaction and ease of use with the G6 System were also assessed.

***Results:*** Data were available from 248 patients (182 with T1D, 66 with T2D; 57% male, 88% non-Hispanic white). Mean (standard deviation) HbA1c fell significantly from 8.2% (1.9%) at baseline to 7.1% (1.1%) at the end of the study (*P* < 0.001); more than half (54.4%) of those with initial HbA1c values >7% experienced absolute HbA1c reductions of >1%. Significant reductions in diabetes distress (DDS) and hypoglycemic concerns (HABS) were observed (*P* < 0.001). Most (93%) participants were satisfied or very satisfied with the G6 System and 73% found it very easy to use.

***Conclusions:*** The first 3 months of CGM use was correlated with improvements in psychosocial outcomes and improved HbA1c levels for people with T1D or T2D who use IIT.

## Introduction

Optimal diabetes management, especially for patients using insulin, requires frequent glucose monitoring, access to infrastructure and support networks, and willingness to adapt to circumstances as they change. Special challenges face people with type 1 diabetes (T1D) because of the absolute requirement for insulin and the need to avoid insulin-induced hypoglycemia, whereas progressive beta cell deterioration in type 2 diabetes (T2D) contributes to the elusive nature of glycemic control.^1^ Most people with diabetes are unable to reach the American Diabetes Association (ADA)-recommended goal^2^ of <7% for glycated hemoglobin A1c (HbA1c), and most people using continuous glucose monitoring (CGM) devices are unable to reach consensus goals^3^ for time in the 70–180 mg/dL target range or time with glucose values >180 mg/dL.^4–6^ Many factors contribute to suboptimal glycemic control, including poor adherence to self-monitoring of blood glucose (SMBG) regimens,^7^ poor adherence to prescribed insulin regimens,^8^ diabetes distress,^9^ and fear of hypoglycemia.^10^ Suboptimal glycemic control contributes to excess morbidity and mortality, negatively influences patients' quality of life (QoL), and adds to the societal costs of diabetes.^1^

Continuous glucose monitoring (CGM) systems offer significant advantages over SMBG-based glucose monitoring regimens^6^ and are foundational to automated insulin delivery systems.^11^ Many of the barriers to CGM adoption imposed by early-generation systems—such as daily calibration, insertion pain, and nuisance alarms—have largely been addressed; modern systems are increasingly available, accurate, and easy to use.^12^

Data from randomized controlled trials indicate that real-time CGM use is associated with QoL and/or glycemic benefits for patients with insulin-treated T1D or T2D.^13–18^ To date, most real-world studies have been limited to anonymized cloud data and lack supportive laboratory tests or robust QoL data.^19–21^ In the Landmark study, we examined glycemic data and QoL outcomes among patients who were using intensive insulin therapy (IIT) to manage their T1D or T2D and began using a real-time CGM system.

## Methods

A real-world prospective study was undertaken in the United States between August 2, 2018, and March 9, 2020. Customer interest was gauged from nationwide callers when they called to place their first Dexcom G6 (Dexcom, Inc., San Diego, CA) order or if they had opted in to receive marketing communication from Dexcom; interested customers were sent invitations and screening questionnaires. Inclusion criteria included ages 25–65 years, use of IIT, no prior CGM use, private insurance, willingness to provide documentation of laboratory or point-of-care HbA1c results and survey responses at baseline and at 12+ weeks after G6 initiation, and willingness to share CGM data by establishing accounts and uploading to the CLARITY web-based portal (Dexcom). Before participating in any study procedures, subjects were asked to voluntarily document their consent by signing an institutional review board (IRB)-approved informed consent form.

Participants who qualified and consented were given access to a study-specific online portal where they uploaded their most recent laboratory or point-of-care HbA1c measurement (taken within the previous 12 weeks) and answered the QoL battery. After 12 weeks, participants returned to the portal to upload a second laboratory or point-of-care HbA1c measurement (taken 12–20 weeks from G6 initiation); complete the follow-up QoL battery; and answer questions about their satisfaction with G6 and its usability. Laboratory or point-of-care HbA1c measurements were verified by study staff. Participants were compensated for completing all surveys and providing the initial and follow-up HbA1c values. Those who had uploaded fewer than 14 days' worth of CGM data over the 12-week study period were excluded from analysis.

QoL questionnaires included the Diabetes Distress Scale (DDS; 17-item scale; 4 subscales; higher scores indicate greater distress)^22,23^ and the Hypoglycemic Attitudes and Behavior Scale (HABS; 14-item scale; 3 subscales).^24,25^ In the DDS, answers to each item are based on a 6-point Likert scale, rated from 1 (“not a problem”) to 6 (“a very serious problem”) for the past month. Subscales evaluate emotional burden (five items), regimen distress (five items), interpersonal distress (three items), and physician distress (four items). The total mean score is calculated and a score of <2.0 is considered as “little or no distress,” 2.0–2.9 as “moderate distress,” and ≥3.0 as “high distress.” In the HABS, answers to each item are based on a 5-point Likert scale (rated from 1, “strongly disagree” to 5, “strongly agree”). The HABS subscales assess participants' hypoglycemia-related anxiety (five items), avoidance (four items), and confidence (five items). Individual domain scores (anxiety, avoidance, and confidence) are calculated by taking the mean score of items in each domain, with higher scores indicating greater concern (anxiety and avoidance domains) or confidence (confidence domain). To produce a total score for the HABS, items in the confidence domain are first reverse scored before the mean score of all 14 items is calculated. Higher total scores indicate greater hypoglycemic concerns.

Paired *t*-tests were performed for comparisons between initial and follow-up HbA1c values and QoL scores using IBM Quantum software.

## Results

A total of 295 individuals enrolled in the study, 248 of whom (182 with T1D, 66 with T2D; 57% male, 88% non-Hispanic white) provided sufficient data for analysis. Participants' mean age was 41.6 ± 10.6 years and was significantly lower for participants with T1D (40.0 ± 10.8 years) than with T2D (46.2 ± 8.9 years) (*P* < 0.05).

**FIG. 1.**
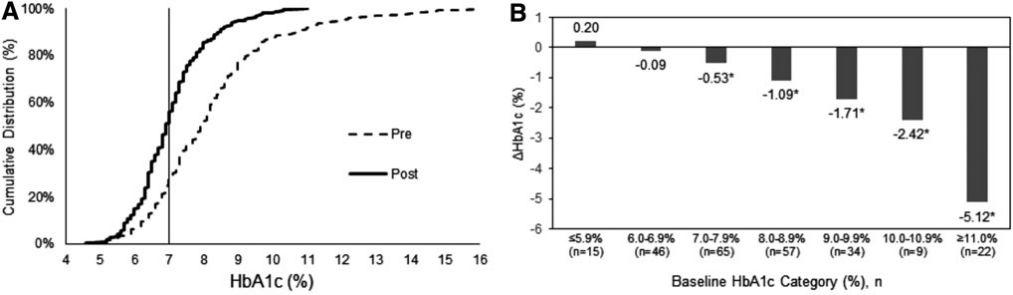
Change in HbA1c during the Landmark study. **(A)** Cumulative distribution of HbA1c levels at baseline (dashed line, “Pre”) and after 12 weeks of rtCGM use (solid line, “Post”). **(B)** Changes in HbA1c according to baseline HbA1c level. *P < 0.001. HbA1c, hemoglobin A1c; rtCGM, real-time continuous glucose monitoring.

Mean ± standard deviation HbA1c level decreased from 8.2% ± 1.9% to 7.1% ± 1.1% (*P* < 0.001) over the study period; statistically significant and clinically meaningful reductions were found in the T1D and T2D subgroups (Table 1). Overall, 79% of participants experienced a decrease in HbA1c. Figure 1 shows the changes in HbA1c levels. The cumulative distributions of HbA1c changes are shown in Figure 1A, where the median (interquartile range) value fell from 7.9% (7.0%–9.0%) before the study to 6.9% (6.4%–7.5%) at the end of the study. The proportion of patients with HbA1c levels of <7% (indicated by the vertical line) increased from 24.6% in the prestudy samples to 50.8% after at least 12 weeks of CGM use. More than half (54.4%) of those participants with prestudy HbA1c >7% experienced absolute HbA1c reductions of >1%. Figure 1B shows that participants with higher HbA1c levels at baseline experienced larger absolute reductions in HbA1c during the study. Of note there were no consistent associations between any of the demographic covariates (age, gender, non-Hispanic white ethnicity, or diabetes type) and change in HbA1c (not shown).

**Table 1.** Comparison of Hemoglobin A1c Values pre- and Post-Continuous Glucose Monitoring Initiation by Participant Diabetes Type

	Overall (*n* = 248)		T1D (*n* = 182)		T2D (*n* = 66)	
	Pre	Post	Pre	Post	Pre	Post
Mean HbA1c (SD), %	8.2 (1.9)	7.1 (1.1)^*^	8.1 (1.7)	7.0 (1.0)^*^	8.5 (2.2)	7.1 (1.1)^*^
Proportion with HbA1c <7.5%	39.5%	73.0%^*^	38.5%	73.1%^*^	42.4%	72.7%^*^
Proportion with HbA1c <7.0%	24.6%	50.8%^*^	25.8%	51.1%^*^	21.2%	50.0%^*^

^*^ *P* < 0.001 versus “Pre” value.

SD, standard deviation; T1D, type 1 diabetes; T2D, type 2 diabetes.

Significant improvements were observed in the total DDS score after 12 weeks of CGM use, for the overall sample as well as for both T1D and T2D subgroups (in all cases, *P* < 0.001). The overall sample and the T2D subgroup demonstrated significant drops in the emotional distress, regimen distress, and interpersonal distress subscales, whereas the T1D subgroup evidenced significant decreases in the emotional distress and regimen distress subscales only (all *P* < 0.001). Similar significant improvement after 12 weeks of CGM use was observed in total HABS scores and all three HABS subscales—for the overall sample and for the T1D and T2D subgroups. Hypoglycemic anxiety and hypoglycemic avoidance dropped significantly for all three groups (*P* < 0.001), whereas hypoglycemic confidence rose for all three groups (though this improvement failed to reach significance for the T2D subgroup; Table 2).

**Table 2.** Mean (Standard Deviation) Scores for Diabetes Distress Scale and Hypoglycemia Attitudes and Behavior Scale and Their Subscales by Participant Diabetes Type

	Overall (*n* = 248)		T1D (*n* = 182)		T2D (*n* = 66)	
	Pre	Post	Pre	Post	Pre	Post
DDS overall	2.46 (0.89)	1.86 (0.72)^*^	2.39 (0.89)	1.86 (0.75)^*^	2.67 (0.85)	1.85 (0.65)^*^
Emotional burden	3.06 (1.24)	2.29 (1.05)^*^	2.94 (1.20)	2.30 (1.07)^*^	3.39 (1.30)	2.26 (1.01)^*^
Physician distress	1.44 (0.79)	1.32 (0.77)	1.48 (0.86)	1.35 (0.81)	1.33 (0.53)	1.25 (0.63)
Regimen distress	2.95 (1.21)	1.91 (0.82)^*^	2.80 (1.17)	1.86 (0.79)^*^	3.36 (1.23)	2.04 (0.91)^*^
Interpersonal distress	2.02 (1.15)	1.75 (0.96)^*^	1.99 (1.14)	1.79 (1.05)	2.12 (1.18)	1.64 (0.65)^*^
HABS overall	2.41 (0.66)	1.97 (0.59)^*^	2.44 (0.67)	1.99 (0.60)^*^	2.32 (0.62)	1.92 (0.55)^*^
Avoidance	2.98 (0.88)	2.37 (0.85)^*^	3.01 (0.88)	2.44 (0.85)^*^	2.89 (0.86)	2.19 (0.81)^*^
Confidence	3.64 (0.83)	4.09 (0.77)^*^	3.62 (0.82)	4.14 (0.68)^*^	3.70 (0.85)	3.96 (0.97)
Anxiety	2.00 (0.80)	1.71 (0.69)^*^	2.04 (0.84)	1.75 (0.70)^*^	1.89 (0.65)	1.60 (0.66)^*^

^*^ *P* < 0.001 versus “Pre” score. For all pre/post comparisons, the directionality of the change indicated an improvement.

DDS, Diabetes Distress Scale; HABS, Hypoglycemia Attitudes and Behavior Scale.

With respect to the consensus goals for percentage of glucose values in various ranges,^3^ 47% of the participants met the goal of having <5% of their values >250 mg/dL, 40% met the goal of having <25% of their values >180 mg/dL, 47% met the goal of having >70% of their values in the 70–180 mg/dL range, 86% met the goal of having <4% of their values in the <70 mg/dL range, and 93% met the goal of having <1% of their values in the <54 mg/dL range. The overall mean glucose level during the study was 161 mg/dL, equivalent to a glucose management indicator^26^ value of 7.2%.

Overall, 93% of participants (93% and 94% of those with T1D and T2D, respectively) were satisfied or very satisfied with the G6 System, and 73% (70% and 80% of those with T1D and T2D, respectively) found it very easy to use.

## Discussion

The Landmark study demonstrated significant glycemic and QoL benefits for first-time CGM use among individuals using IIT to manage either T1D or T2D. After ∼12 weeks of Dexcom G6 use, participants had a mean absolute reduction in HbA1c levels of 1.1%, and more than half of those with initial HbA1c values >7% experienced absolute HbA1c reductions of >1%. The reduction in HbA1c observed in Landmark was similar for patients with T1D and T2D and was more pronounced for participants with higher baseline HbA1c, consistent with observations from the DIAMOND randomized controlled trial.^27^ In the Landmark study, there was no standardized training or intervention at CGM initiation, suggesting that the glycemic benefits can be realized without formal instruction.

To our knowledge, this is among the first studies to evaluate QoL prospectively using validated questionnaires in a large real-world population of patients on IIT to manage their diabetes. After 12 weeks, both diabetes distress and hypoglycemic concerns dropped significantly, regardless of diabetes type. This is distinct from the data published in the DIAMOND study, where changes in diabetes distress were observed for participants with T1D, but not for those with T2D.^15,18^ However, baseline DDS scores were much higher in Landmark than in DIAMOND; also, CGM feature-set improvements have been made since DIAMOND.

A principal strength of the study is that it represents real-world outcomes among people new to CGM with a wide range of baseline HbA1c values. Principal limitations include the lack of a control group and the absence of baseline blinded CGM data, which prevents a meaningful assessment of changes in CGM-based metrics of glycemic control. Other limitations for the study include the possible heterogeneity in HbA1c measurement method (point-of-care vs. laboratory reporting), a lack of information about comorbidities that might affect the relationship between ambient glucose and HbA1c, and lack of information about insulin delivery modality; some participants may have used G6 to drive hybrid-closed loop systems (like Control-IQ), which could have contributed significantly to HbA1c improvements.^11^ In addition, although the G6 CGM System offers several optional features whose use is associated with favorable glycemic outcomes,^28^ we did not monitor their use in this study. Furthermore, the results may not be generalizable to users of other real-time or intermittently scanned CGM systems; to patients younger than 25 years, older than 65 years, or not on IIT; or to patients who are not actively engaged in managing their diabetes. Last, the study stopped enrolling at the start of the COVID-19 pandemic and we cannot comment on the influence of the pandemic on changes in HbA1c. However, we expect the effect of the pandemic to have been modest, given changes in mean glucose reported by Van der Linden et al.^29^ in this same issue.

From the Landmark data presented here, we conclude that the first 3 months of real-time CGM usage by patients with intensively managed diabetes is associated with meaningful improvements in HbA1c and QoL. Further studies that evaluate long-term outcomes among CGM users are warranted, as are studies of technology-driven diabetes management strategies as they become available. Such studies are likely to provide insights into how CGM and related technologies can reduce patient burden and regimen complexity, potentially delay the need for pharmacotherapy intensification, and improve the quality of care.
